# High performance wash-free magnetic bioassays through microfluidically enhanced particle specificity

**DOI:** 10.1038/srep11693

**Published:** 2015-06-30

**Authors:** Daniel J.B. Bechstein, Jung-Rok Lee, Chin Chun Ooi, Adi W. Gani, Kyunglok Kim, Robert J. Wilson, Shan X. Wang

**Affiliations:** 1 Department of Mechanical Engineering; 2 Department of Chemical Engineering; 3 Department of Electrical Engineering; 4 Department of Materials Science and Engineering. Address for all: Stanford University, 476 Lomita Mall, Stanford, California 94305, USA

## Abstract

Magnetic biosensors have emerged as a sensitive and versatile platform for high performance medical diagnostics. These magnetic biosensors require well-tailored magnetic particles as detection probes, which need to give rise to a large and specific biological signal while showing very low nonspecific binding. This is especially important in wash-free bioassay protocols, which do not require removal of particles before measurement, often a necessity in point of care diagnostics. Here we show that magnetic interactions between magnetic particles and magnetized sensors dramatically impact particle transport and magnetic adhesion to the sensor surfaces. We investigate the dynamics of magnetic particles’ biomolecular binding and magnetic adhesion to the sensor surface using microfluidic experiments. We elucidate how flow forces can inhibit magnetic adhesion, greatly diminishing or even eliminating nonspecific signals in wash-free magnetic bioassays, and enhancing signal to noise ratios by several orders of magnitude. Our method is useful for selecting and optimizing magnetic particles for a wide range of magnetic sensor platforms.

Ongoing advances in magnetic biosensing technologies over the last decade have led to a huge boost in their performance^1^,^2^,^3^,^4^,^5^,^6^,^7^,^8^,^9^,^10^,^11^,^12^,^13^,^14^. Employing magnetic biosensors that detect magnetically labeled biomolecules, these technologies are pushing the limits of protein detection to lower detection thresholds^5^,^14^,^15^,^16^,^17^,^18^ and correspondingly enable earlier disease detection in medical diagnostics. A variety of commercial magnetic micro- and nanoparticles are available as magnetic labels for biosensor use^5^,^14^,^19^,^20^. It is well known that a magnetic particle’s size and magnetization characteristics heavily influence the signal from magnetic sensors that detect local magnetic field changes. Larger and more magnetizable particles generally yield a higher signal per particle.

To optimize a detection system, it is necessary to consider not only the sensor and the particle by themselves, but also the whole system Signal to Noise Ratio (SNR). For optimal SNR, the specific binding signal needs to be large, while the noise (nonspecifically adhering particles and electronic noise) needs to be small. For the specific signal, in addition to the particle’s magnetic properties, both particle transport to the surface (diffusion and convection are both heavily particle size dependent) and particle-to-sensor binding need to be considered^21^. For the non-specific binding signal, effects from nonspecific binding of biological, chemical, electric and magnetic origin need to be considered. While biological and chemical effects depend on the analytes and reagents as well as functionalization, electric and magnetic effects are more fundamental to the sensor system. The electrostatic forces between particle and sensor are attractive or repulsive as a function of zeta potentials of sensors and particles. Generally, particles that have a slightly negative zeta potential are subject to a small repulsive force from slightly negatively charged sensor surfaces, thus significantly reducing nonspecific adhesion to the sensor^22^ and improving the SNR.

We have demonstrated highly sensitive detection of proteins, DNA, and other biological analytes using a magnetic sandwich assay format on our Giant Magnetoresistive (GMR) biosensor platform^7^,^8^,^15^,^16^,^23^,^24^. In all of our recently published work on GMR biosensors, we have used sub-micron sized magnetic nanoparticles of nominal diameter 50 nm (Miltenyi MACS) which are relatively small compared to commonly used micron sized (and larger) magnetic particles^9^,^18^,^25^,^26^,^27^,^28^,^29^. When magnetized by an applied AC magnetic field which enables sensor readout, these nanoparticles showed good specific binding characteristics, while nonspecific adhesion or settling onto the sensor surface was undetectable. GMR sensors can be engineered to only sense particles within hundreds of nm proximity to sensor surfaces, due to the inverse cubic distance dependence of the particles’ decaying magnetic dipole field. Indeed, unbound magnetic particles in solution above the sensor surface do not contribute to the signal at the particle concentrations we employ for any of the particles studied. Therefore, this proximity sensing approach allows us to record accurate real time binding curves without the need for washing away the unbound particles from the open reaction well above the sensor^23^ (supplemental Figure S1). Compared to optical methods requiring the washing of reagents at a discrete time point before readout, this magnetic wash-free assay method does not entail any tradeoff between assay speed and accuracy. Instead, it delivers both through its continuous sampling^24^,^30^.

In our previous attempts to increase the signal in our wash free assay, larger (but still sub-micron sized) magnetic particles have failed in open well assays due to particle adhesion on the sensor. This adhesion to the magnetized sensor has caused large background signals, almost indistinguishable from the signal of interest, and thus has hugely decreased the SNR compared to that of smaller particles. The adhered particles could be removed in a washing step after assay completion that aspirates unbound particle solution and replaces it with a washing buffer. While the washing step in our open well assay format has significantly reduced the nonspecific adhesion of larger magnetic particles, washing did not completely eliminate the adhesion and did not yield reproducible results, possibly due to extreme flow non-uniformity during crude open well washing. Additionally and more importantly, the introduction of a washing step defeats the purpose of a wash-free assay by allowing accurate measurements only after completed washing, eliminating desirable time resolved binding data. This need for washing is also a major drawback for many magnetic sensor platforms employing micron sized and larger particles^14^. These platforms generally do have the problem of nonspecific binding and adhesion of particles to the sensor surface and have to introduce strategies to reduce nonspecific binding and enhance assay sensitivity. Fluid force discrimination is one strategy to remove nonspecific bound particles with a laminar flow over the sensor surface^25^,^26^.

Our failed endeavors to use larger submicron magnetic particles appeared to be at odds with another study of submicron magnetic particle to surface binding and adhesion which found negligible nonspecific binding and adhesion^22^. However, in the latter work, the particle-sensor system was simplified with particle binding on a functionalized nonmagnetic surface (gold surface in surface plasmon resonance experiments) and thus only biological, chemical and electrical effects were considered. Since the study neglected magnetic interactions between particles and sensor in the presence of an applied field, it could not explain the non-specific adhesion of magnetic origin of these larger particles observed on our GMR sensor, which were not present for small MACS particles.

Thus, there is a need for a thorough investigation to elucidate the influence of the particles’ magnetic adhesion. Understanding and inhibiting this non-specific magnetic adhesion could allow the introduction of larger magnetic particles in wash-free magnetic immunoassays, thereby providing higher SNR. Therefore it is sensible to analyze the particle adhesion in the most realistic model system – the sensor itself in the presence of an applied magnetic field, which is necessary to magnetize the superparamagnetic particles for magnetic signal transduction. Since magnetic adhesion forces tend to be weak and particles can be released through washing, these forces could instead be overcome by applying a flow over the sensor area in a reproducible and uniform fashion. Instead of a separate washing step used in fluid force discrimination 25,26, which is not compatible with the wash-free approach, a microfluidic assay format was chosen that allows precise control and repeatability of the applied viscous flow forces to selectively remove only nonspecifically adhered particles at the same time as delivering analyte and reagents.

Here we investigate the magnetic adhesion of a selection of commercially available magnetic particles on magnetic sensors in an applied field. We expand our wash-free assay technique to a microfluidic format and measure the magnetic particles’ time-resolved biomolecular binding and magnetic adhesion dynamics. Specifically, we flow magnetic particles at constant flowrate over the magnetic sensor surface using microfluidic channels and acquire magnetic sensor data measuring both specifically binding and nonspecifically adhering particles. The biosensor’s real time readout gives instantaneous feedback of the particle’s binding and adhesion dynamics at the given flowrate. By adjusting the particle solution flowrate, we can dynamically probe the particle’s binding characteristics and thus can quickly optimize the flowrate for highest SNR. To probe the binding mechanism of magnetic particles at sensor surface, we then observe and compare binding location distributions of different magnetic particle types in Scanning Electron Microscope (SEM) images of the bound particles. We show the influence of the magnetic forces responsible for the magnetic adhesion and quantify these with magnetic simulations that support our experiments. We then demonstrate that in wash-free microfluidic bioassays the diameter of suitable magnetic particles and SNR can be greatly increased, enabling larger particles that suffered deleterious adhesion in open-well assays. Finally, using the newly enabled larger magnetic particles, we design and obtain reproducible wash-free microfluidic magnetic bioassays. These yield a tenfold improvement in signal magnitude and an order of magnitude lower protein detection threshold.

## Results

**Figure 1:**
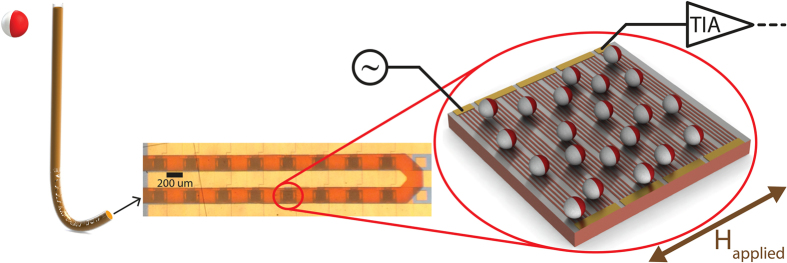
Illustration of magnetic particle based sensing using microchannels for particle delivery. Magnetic particles are flowed from a sample tube (left) using a syringe pump through microchannels (middle - 200 um width, 50 um height) over the GMR sensor surface and are captured on the sensor surface (right – illustration not to scale). Each GMR sensor consists of a series connection of 8 bundles of 11 parallel connected GMR strips, which are connected to a transimpedance amplifier (TIA) and subsequent data acquisition electronics^8^ that measures the resistance change due to the presence of nanoparticles in an applied magnetic field (H_applied_).

Streptavidin coated magnetic nanoparticles are flowed through microfluidic channels over the sensor chip using our microfluidic giant magnetoresistive (GMR) sensor setup (Fig. 1). The particles come into contact with differently coated sensors. Bovine Serum Albumin (BSA) coated sensor surfaces act as controls for biologically nonspecific adhesion (non-targeted sites) and Biotin-BSA coated sensor surfaces allow specific binding via the streptavidin-biotin bond (targeted sites).

**Figure 2:**
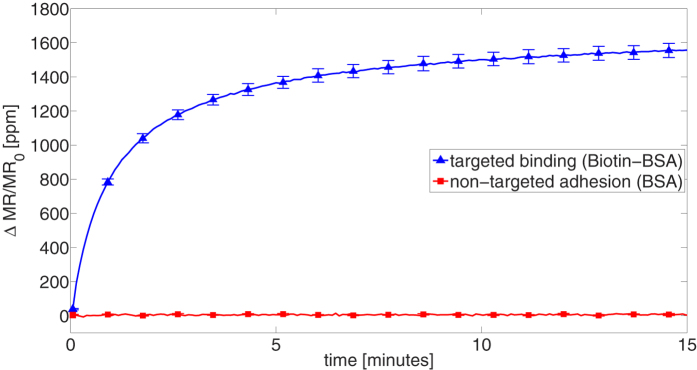
Real time binding and adhesion curves for streptavidin-coated MACS (50 nm diameter) particles. Real time binding curves are acquired for targeted binding to biotin-BSA coated sensors (blue curve) or adhering to non-targeted BSA coated sensors (red curve). Particles are delivered through microchannels at constant flowrate of 2 uL/min. Error bars denote sample standard deviation of replicate sensors on the same GMR chip.

Using this setup, we record both targeted biomolecular binding (sum of specific binding and magnetic adhesion) and non-targeted adhesion (sum of nonspecific binding and magnetic adhesion) in real time (Fig. 2) at a constant flowrate for streptavidin-conjugated MACS particles. The sensor signal (normalized magnetoresistance change in ppm) from targeted binding rises when particles hit the sensor surface. The signal reaches its temporal asymptotic value within 10-15 minutes due to our choice of streptavidin coated MACS particles – a timescale equal to open well assays without microfluidics^24^. The non-targeted adhesion signal is close to zero, indicating that for MACS particles both nonspecific binding and magnetic adhesion are very low.

**Figure 3:**
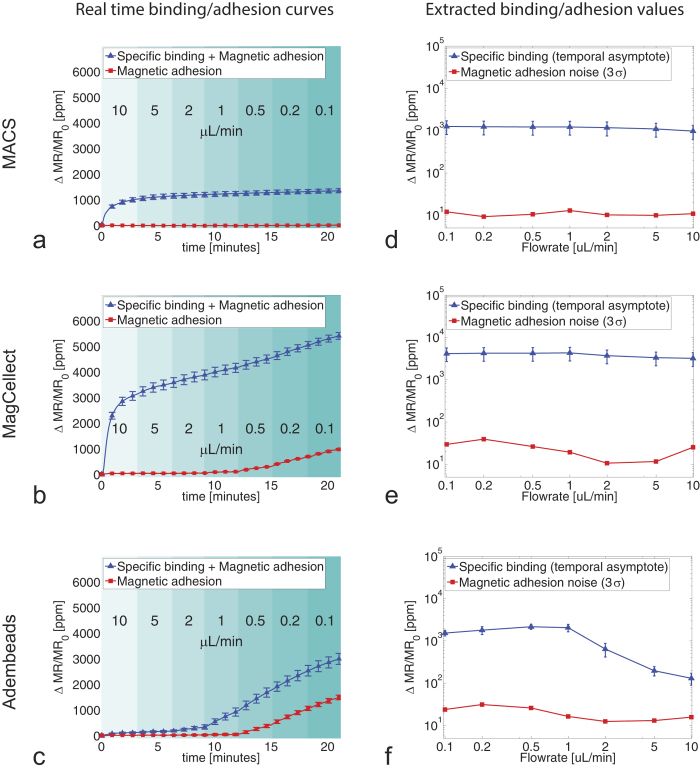
Specific binding and magnetic adhesion for 3 differently sized magnetic particles as a function of time varying flowrate. Real time binding curves are acquired for streptavidin-coated magnetic particles of different sizes: (**a**) Miltenyi MACS (50 nm, *c* = 2 · 10^12^ particles/mL), (**b**) RnD MagCellect (150 nm, *c *= 6 · 10^10^ particles/mL), (**c**) Ademtech Bio-Adembeads (300 nm, *c *= 1.2 · 10^10^ particles/mL). The particles get captured either by biotin-BSA coated sensors (combination of specific binding and magnetic adhesion, blue curves) or by BSA coated sensors (magnetic adhesion only, red curves). Flowrates are changed about every 3 minutes (indicated in different shading). The specific binding portion (temporal asymptotic value of the exponential fit of the difference between blue and red curves in **a**,**b**,**c**) and a magnetic adhesion noise estimate (three times standard deviation of slope-removed data), both extracted from (**a**,**b**,**c**) are plotted in (**d**,**e**,**f**). All data is acquired in parallel microfluidic channels on the same GMR sensor die. Error bars denote sample standard deviation.

Next, we extend this experiment to a range of flowrates and a selection of particles. We dynamically change the flowrate in the experiment, while recording sensor responses continuously (Fig. 3). The flowrate starts at a high value of 10 uL/min and is about halved every 3 minutes until it reaches the minimum value of 0.1 uL/min. We extract the temporal asymptotic value and use that to quantify the targeted particle binding (see methods and supplemental Figure S7).

The particles in the 50–300 nm diameter range (MACS, MagCellect, Bio-Adembeads) gave rise to a specific binding signal. Variations in signal for different particles on sensors with no biological specific binding indicated that nonspecific binding was dominated by magnetic adhesion for the higher moment particles. While we characterized particles in the range between 50 and 300 nm, we also tested even larger particles (500 nm Adembeads, 1 micron Dynal beads). However, in the presence of the applied magnetic field, these larger particles coagulated to a big clump that could not enter the microfluidic chip. Therefore these larger particles could not be analyzed further in this study.

Clearly the variably sized particles show very different binding characteristics, which are especially flowrate-dependent for the larger tested particles. MACS (50 nm) binding characteristics showed negligible magnetic adhesion and a constant specific binding signal over the whole flow range. The larger MagCellect particles (150 nm) showed small magnetic adhesion at flowrates above 1 uL/min, with an onset of magnetic adhesion below this value. The specific binding signal significantly increased compared to MACS, and was only slightly dependent on the flowrate. The even larger Adembeads particles (300 nm) showed small magnetic adhesion at flowrates above 1 uL/min. Adembeads’ ability to reach the sensor surface was limited by diffusion for flowrates of 2 uL/min and above, leading to small specific binding signals. This yields only a very small suitable flowrate window.

The SNR can be estimated by dividing the specific signal by the noise estimate (see supplemental Figure S2). That translates into a constantly high SNR for MACS over the range of flowrates, and an SNR that peaks at 2 uL/min for both MagCellect and Adembeads particles. MagCellect particles at 2 uL/min show the highest SNR of these experiments.

Once specifically bound via the strong biotin-streptavidin bond, none of the tested particles unbind at flowrates up to 20 uL/min (highest flowrate tested). At these flowrates, particles nonspecifically adhering to BSA Biotin sensors are released (data not shown here).

**Figure 4:**
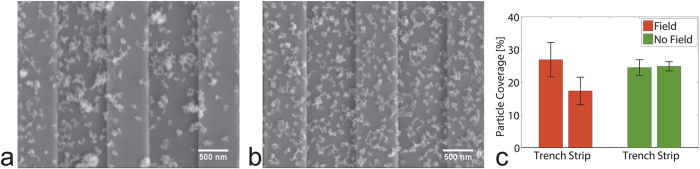
Scanning Electron Microscope images of magnetic nanoparticles (bright spots) after specific binding to a biotin functionalized sensor area. The sensor chip in (**a**) was subject to an applied magnetic field, (**b**) was without applied magnetic field, both with magnetic particles flowed over the sensors at a constant flowrate of 2 uL/min. Trenches (800 nm wide, darker background) are located between the sensor’s parallel spin valve strips (600 nm wide). In (**a**) MagCellect particles are preferentially located in trenches, whereas in (**b**) MagCellect particles are uniformly distributed over trenches and strips. (**c**) From 2 sets of SEM Images, particle coverage ratios are extracted and plotted individually for trenches and strips. Error bars denote sample standard deviation of multiple locations (n = 4 for trench, n = 6 for strip). SEM images of MACS and Adembeads are in supplemental Figure S3.

After the experimental runs were finished, SEM images of the bound particles on the individual sensors were acquired and compared to a control experiment without applied magnetic field (Fig. 4).

For particles subject to the applied magnetic field, the location of the specifically bound particles was strikingly different in terms of being atop the sensor strip vs. being in a trench between strips. MACS particles bound on both strips and trenches, MagCellect particles bound preferentially in trenches and Adembeads particles bound almost exclusively within trenches (Fig. 4a and supplemental Figure S3). The experiments without magnetic field showed no preference for either strips or trenches for all particles tested (MACS, MagCellect, Adembeads). Additionally a much lower particle density for Adembeads particles was observed in experiments without magnetic field compared to experiments with magnetic field (Fig. 4b and supplemental Figure S3). Particle coverage ratios are extracted (supplemental Figure S4). For magnetically adhering particles the recorded binding and adhesion curves (Fig. 3) give a much more accurate measure than SEM images, since SEM images can only be accurately taken after rinsing the washing buffer to avoid the formation of salt crystals, which then also washes away many of the magnetically adhering particles.

This binding location is known to have a big impact on the sensor signal. Particles are magnetized by the total magnetic field, which is a superposition of externally applied field and the stray field of magnetized magnetic sensor. Inside the GMR sensing layer the applied field is mostly parallel to the dipole field of particles binding within the trenches, and mostly antiparallel to the particles on the sensor strips. This parallel magnetization yields a positive signal for particles located in the trenches and particles on the sensor strip close to its edge. Conversely, it yields a negative signal for locations above the sensor strip (supplemental Figure S1). Thus a non uniform binding of the particles might alter the signal. Particles with a strong binding preference to the trenches might yield an enhanced signal.

### Magnetic Force Simulations

**Figure 5:**
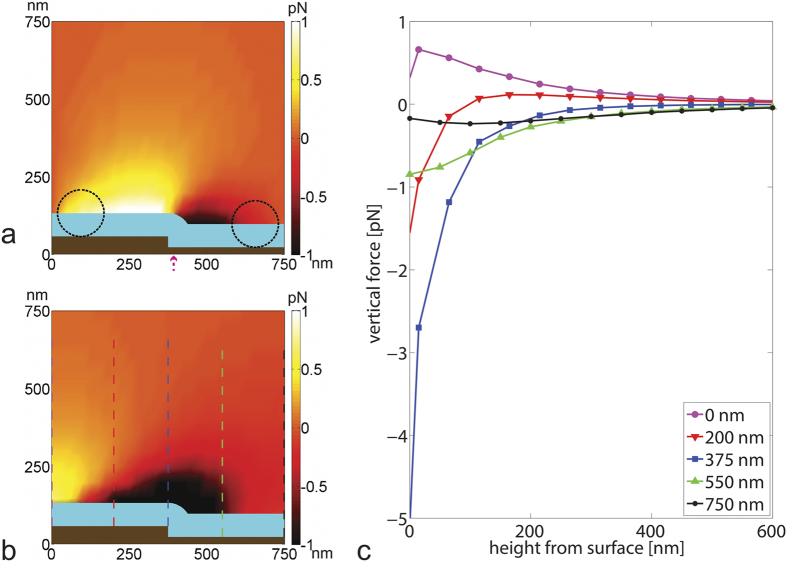
Plots of (**a**) horizontal and (**b**,**c**) vertical magnetic forces acting on MagCellect nanoparticles. Forces are plotted in a cross-section of half of a unit sensor strip / trench cell (axisymmetric to the left and the right plot border). The sensor strip and trench region (including all magnetic and passivation layers) are colored brown. The vertical direction is perpendicular to the sensor surface and the positive horizontal axis is along the flow direction. Particles are modeled as Langevin spheres and the force is calculated at the center of the particle (details in Methods). The finite particle size leads to forbidden particle locations - one particle radius or less away from the sensor - colored light blue. An AC magnetic field is applied, so the magnetization of the magnetic nanoparticles and of the GMR sensor change over time. Calculated forces for maximum magnetic field amplitude (for 50 Oe) are shown here, with the time-averaged force at half this value. Positive values denote forces in positive x (in horizontal plot) or y direction (in vertical plot). (**a**) The horizontal magnetic forces for MagCellect particles are on the order of 1 pN. Opposing horizontal forces push particles from both directions towards the trench edge. The zero force equilibrium point for the horizontal magnetic force is not directly at the sensor edge but moved from there into the trench (see arrow under image). Dotted circles are shown for particle size comparison. (**b**) The vertical forces on the particles are attractive above the trench (with particles magnetized the same direction as the field gradient) and repulsive over the sensor strip close to the sensor surface (with particles magnetized by the applied field which is opposing the direction of the sensor stray field gradient – see supplemental figure S1). (**c**) Approach curves show consistently attractive forces for particles close to (green curves) or at (blue curves) trench edge but a repulsive force barrier of about 0.4 pN over a distance of 200 nm for particles approaching over the sensor stack (red curves).

In magnetic simulations, the relevant in-plane and perpendicular magnetic forces exerted by the sensor on the different sized magnetic particles (by center point location) are calculated and shown in a force field plot (Fig. 5).

Vertical and horizontal forces acting on Adembeads are qualitatively and in magnitude the same as on MagCellect particles and forces on MACS are about 2 orders of magnitude lower (see supplemental Figures S5).

For the vertical force, the repulsive region above the sensor strip is consistent with SEM results of the larger particles which show significantly inhibited binding on the sensor strip.

The zero force equilibrium point is located not at the trench edge, but offset slightly into the trench. The reason for the offset is the finite size of the particle that is subject to the magnetic field over its entire volume. As such, this offset increases with larger particle diameters: it is negligible for the MACS particles, 25 nm into trench for MagCellect particles and 60 nm into trench for Adembeads particles. This offset of the equilibrium point into the trench is consistent with the SEM results that favor trench regions as particle locations for Adembeads and MagCellect particles under applied field.

The size of magnetic nanoparticles is an important factor for particle’s magnetic moment and forces since larger particles generally have larger magnetic moments. However, it is important to note that the commercial particles evaluated also varied in their composition and magnetic properties, which also heavily influences the particles’ magnetic moments (supplemental Table S1). The magnetic susceptibility therefore needs to be considered along with the size to quantify a magnetic particle’s interaction strength. Here the MagCellect particles have a more than 3 times larger magnetic susceptibility (χ) as compared to the Ademtech particles (see supplemental Table S1). The increased susceptibility partly compensates for the smaller size (factor 8 in volume) and thus leads to roughly comparable magnetic forces.

### Biological assay experiment

**Figure 6:**
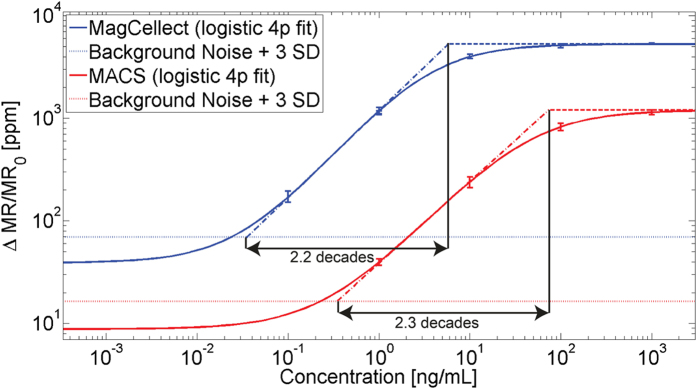
Standard curves translating a range of C-reactive protein concentrations to a magnetoresistive signal in magnetic immunoassays using either MagCellect or MACS particles. Standard curves are extracted from serial factor of 10 dilutions in this immunoassay (1 ug/mL as highest concentration) averaging 8 individual data curves per concentration and particle. The detection threshold (crossing the background + 3 SD level) is 225 pg/mL for MACS and 23.7 pg/mL for MagCellect particles. Error bars denote sample standard deviations (n = 8) and are only shown above the detection threshold.

With the observation of high SNR in both MagCellect and MACS particles, and much improved signal strength in MagCellect particles, we compare the two particles’ performance in a magnetic immunoassay experiment. In this assay format, the sensor surface is functionalized with capture probes that bind to the target analyte of interest. The target antibody is in turn bound again by a secondary biotinylated detection probe, which is ultimately labeled with a streptavidin-coated magnetic nanoparticle^16^. We use a slightly modified microfluidic setup from Fig. 1 allowing us to first flow protein analyte, the detection antibody, and then the magnetic particles over the capture antibody pre-functionalized GMR sensor surface at 2 uL/min. For each concentration and particle we record 8 data curves. We use the saturation levels (after having flowed magnetic particles for 15 min) to plot a standard curve for both particle types: MagCellect and MACS (Fig. 6). The demonstrated biological assay measures the concentration of C-reactive protein (CRP). CRP is used as an inflammation biomarker^31^.

MagCellect particles reduce the analyte detection threshold by one order of magnitude and allow detection of 23.7 pg/mL of CRP in these experiments. Using MACS particles under the same conditions required 225 pg/mL of CRP to be detected. Both assays have comparable dynamic range of 2.2 and 2.3 decades (orders of magnitude).

## Discussion

In our earlier publications we routinely used only MACS magnetic particles in bioassays, which did not show magnetic adhesion to the magnetized sensor. We thus previously assumed that magnetic interactions between particles and sensor were negligible. Here, we have resolved the magnetic adhesion dynamics and have shown that the magnetic interactions between particles and magnetized sensors can not generally be neglected.

None of the tested particles adhered nonspecifically to the magnetic sensor surface without applied magnetic field, which is consistent with other particle binding experiments^22^. However, applying a magnetic field, which is necessary for sensor operation, changes this situation. MagCellect and Adembeads particles are magnetically captured and adhere on the surface, provided the shear flow drag force is less than an observed threshold. While most magnetically adhered particles could be washed away upon increasing the flowrate, some still adhered to the sensor surface. However, flowrates larger than 1 uL/min severely inhibit the specific binding for the Adembeads particles and slightly reduce it for MagCellect particles.

To overcome horizontal magnetic trapping of a MagCellect particle held with magnetic force of 1 pN, a flowrate of 20 uL/min would be needed to generate a balancing drag force of 1 pN at steady state. While the flow force is acting continually on the particles, the magnetic force, deriving from the alternating magnetic field, is sinusoidally modulated. Furthermore the horizontal forces are location dependent, thus once the particle is moved through the high field gradient region, lower magnetic forces will act on the particle. Taking into account this time and space dependent magnetic force (and not just its temporal and spatial peak), yields to a decrease of the required drag force or equivalent flowrate, which is consistent with the 2 uL/min (and equivalent 0.1 pN drag force) threshold observed for MagCellect particles.

Thus using optimized flowrates, we achieved greatly enhanced SNRs in the range of 10^2^–10^3^. These SNR levels show a several order of magnitude enhancement compared to Ademteads and MagCellect particles in open well assays without flow, where the specific binding and nonspecific adhesion signal levels are comparable to each other (see supplemental Figure S6).

Modifying and optimizing the surface properties of the sensor and particle could allow a better electrostatic repulsion of particles from sensor and thus reduce the nonspecific binding even more. Additionally, a reduction of the duty cycle of the magnetic readout field (no continuous sampling but only at a low percentage of the time), and thus not applying the magnetic force continuously, would limit the magnetic adhesion further.

We demonstrated that careful control of the flowrate limits magnetic adhesion. Limiting magnetic adhesion using optimized flowrate, we extended our wash-free assay approach with real-time readout to MagCellect particles. We achieved a one order of magnitude lower protein detection threshold of MagCellect vs. MACS magnetic nanoparticles through judicious use of microfluidic flow that limited magnetic adhesion.

We found that the binding location of magnetic particles differs notably with particle size. Magnetic simulations confirmed that vertical and horizontal magnetic forces increase in magnitude with particle size. Vertical forces show an attractive region above the trench and edge, while horizontal forces push particles towards an equilibrium location close to the edge. This binding location preference vanishes when no magnetic field is present. Binding locations from SEM images are consistent with the simulation results and confirm that the magnetic force cannot be neglected. The magnetic force acting on a magnetic particle pulls it to the sensor surface and can cause magnetic adhesion of particles on the sensor.

In conclusion, we demonstrated that magnetic interactions between sensors and magnetic particles are large enough to impact particle transport and adhesion of magnetic particles to magnetic sensors. The easily adjustable applied fluidic force provides a fast mechanism to probe particle’s specific binding and magnetic adhesion dynamics. Our system can quickly and easily characterize the binding and adhesion of magnetic particles directly on a sensor under an applied magnetic field. This method can be easily modified and employed to characterize all kinds of magnetic particle and sensor system combinations.

## Methods

### Magnetic Sensor Array

The sensor array comprises 64 individual spin valve type Giant Magnetoresistive (GMR) sensors arranged in an 8 by 8 sensor array^16^. The active sensor area of each individual sensor is 100 by 130 um, and the 64 sensor array occupies a total area of 3300 um by 2900 um. An alternating magnetic field (0.005 T, f = 210 Hz) magnetizes the nanoparticles. The sensor transforms the nanoparticle-induced magnetic field change into a resistance change, which can be electrically read out. A double modulation scheme using both a sinusoidally modulated magnetic field (210 Hz) and electric readout current (540 or 590 Hz) is employed for faster readout of the entire 64 sensor array. AC voltages with two distinct frequencies are applied simultaneously, one (540 Hz) to rows 1,2,5,6 and the other (590 Hz) to rows 3,4,7,8, while all 8 sensors per row are concurrently read out using a custom data acquisition board. A LabVIEW program interfaces the data acquisition board and includes a temperature correction scheme that eliminates temperature-induced signal drift^8^. One readout cycle of the whole array takes 5.26 s.

### Particles

The following streptavidin-coated magnetic particles were used in the experiments: Miltenyi Biotec μMACS Streptavidin MicroBeads, RnD Systems MagCellect Streptavidin Ferrofluid (MAG999), and Ademtech Bio-Adembeads Streptavidin plus 300.

### Binding and adhesion experiments with microfluidic flow

Biotin-BSA and BSA are spotted (1.2 nL per individual sensor) using a Scienion Flexarrayer S5. In addition, passivated electrical reference sensors are not spotted, and are shielded from the environment by a thick oxide layer, and thus not sensitive to magnetic particle above the oxide layer. Syringe tubes are loaded with streptavidin-coated superparamagnetic nanoparticles, one tube per type of particles. Using a syringe pump system (NE-1800, New Era Pump Systems), the particles are injected into the microfluidic chip inlet and flowed over the sensors at either a constant flowrate of 2 uL/min (Experiment of Fig. 2) or at varying flowrates (Experiment of Fig. 3).

### Microfluidic chip

The microfluidic chip is fabricated based on standard PDMS microfluidic chip soft lithography fabrication processes molded from SU-8 channel negative^32^ and has a rectangular channel profile (width 200 um, height 50 um). Before use, the chip is flushed with Pluronic F-68 (4%) and incubated for 30 min. The microfluidic chip directly interfaces the biosensor array with its opening void.

### SEM data analysis

The SEM data analysis was performed with Image J. Individual trench and strip sections were background subtracted and the threshold automatically detected. Particle coverage was then measured as the percentage of bright pixels (particles) above the set threshold.

### Magnetic Simulation and modeling

Particles are modeled as Langevin spheres in a field regime where the magnetic response is linear, since the applied magnetic field is much lower than the particle saturation field. Particle magnetic properties are obtained from magnetic moment measurements of sample volumes in the 10–25 uL range, and dividing by the number of nanoparticles. The nanoparticle number densities were measured (using optical tracking and counting) using a Nanosight tool. The magnetic properties are in Supplemental Table S1.

Ansoft’s Maxwell magnetic simulation software is used to simulate the magnetic fields present in the magnetized GMR system. Field values are simulated for half a unit cell (sensor strip and trench) for an infinitely long sensor (actual aspect ratio is about 1000) subject to the applied magnetic field. As all particles are in the linear regime, their magnetization is only dependent on the field, the particle’s size and susceptibility. The magnetic force acting on them can be calculated by integration over the spherical volume of the particle: *F*_*mag*_ = 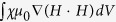. The forces are then plotted at the centerpoint of each sphere.

### Immunoassay

The presented magnetic immunoassay experiment is similar to our open well magnetic immunoassays, but with the additional use of microfluidic sample and reagent delivery. We flow reagents through the microfluidic chip over the sensor similar to the binding and adhesion experiments. Capture antibodies are immobilized along with Biotin-BSA and Biotin controls on different individual sensors using a Scienion Flexarrayer S5. A syringe tube is loaded with 30 uL CRP (concentrations ranging from 10^−3^ ng/mL to 10^3^ ng/mL), 30 uL biotinylated anti-CRP detection antibody (2 ug/mL) and 15 uL streptavidin-coated superparamagnetic nanoparticles (depending on the assay either Miltenyi Biotec MACS at 2 · 10^12^ particles/mL, or RnD MagCellect at 6 · 10^10^ particles/mL, details in Supplemental Table S1), each separated by an air bubble. Using the syringe pump system, the reagents are sequentially injected into the microfluidic chip inlets at a constant flowrate of 2 uL/min and flowed over the sensors.

### Asymptote extraction

To obtain the specific binding signal, we subtract the BSA signal (magnetic adhesion) from the Biotin-BSA signal (specific binding + magnetic adhesion). Afterwards, to extract the temporal asymptotic value from the specific binding signal (from Fig. 3), we use MATLAB to curve fit each segment of the data for each (~3 min) flowrate window with an exponential function 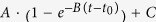 with fitting parameters *A*,*B* and *C*, time variable *t* and start time *t*_0_. The temporal asymptotic value is A + C. Supplemental Figure S7 illustrates this fitting method.

### Data processing and statistical analysis for Immunoassay

A plane is fitted through the 4 electrical reference sensor signals (in the 8 × 8 sensor array) and is subtracted from the active sensor signals for each time point. Error bars denote sample standard deviations and are calculated for each group of sensors (12 active protein sensors each per channel, 6 positive control, 6 negative control, 4 electrical reference). For standard curves an optimized flowrate of 2 uL/min is used, amplitudes are extracted from the mean signal of the time points between 10 and 15 mins after rise of the positive control signal. The BSA background level (signal + 3 SD) showed same values as the zero analyte signal. The standard curves are fitted with a 4 parameter logistic function.

## Additional Information

**How to cite this article**: Bechstein, D.J.B. *et al.* High performance wash-free magnetic bioassays through microfluidically enhanced particle specificity. *Sci. Rep.* **5**, 11693; doi: 10.1038/srep11693 (2015).

## Supplementary Material

Supplementary Information
