# Supplementary material for: High performance wash-free magnetic bioassays through microfluidically enhanced particle specificity
Source: Sci Rep. 2015 Jun 30;5:11693. doi: 10.1038/srep11693 (PMC4485157; doi:10.1038/srep11693)
Supplement: Supplementary Information [file srep11693-s1.pdf]

Supplemental Info for: High performance wash-free magnetic bioassays through microfluidically enhanced particle specificity

Daniel J.B. Bechstein[1], Jung-Rok Lee[1], Chin Chun Ooi[2], Adi W. Gani[3], Kyunglok Kim[3], Robert J. Wilson[4] and Shan X. Wang[3,4]

1 Department of Mechanical Engineering, 2 Department of Chemical Engineering, 3 Department of Electrical Engineering, 4 Department of Materials Science and Engineering. All: Stanford University, 476 Lomita Mall, Stanford, California 94305, USA

GMR sensor signal sign due to magnetic particle

The following illustration (Figure S1) schematically illustrates the sensor signal change due to a single particle. Sensor and particle are magnetized by a sinusoidally alternating magnetic field. At any time point the magnetic field magnetizes the sensor's sensing layer. Particles are magnetized by the total magnetic field, which is a superposition of externally applied field and the stray field of magnetized magnetic sensor. Due to the low frequency (210 Hz) there is no phase difference between the sinusoidally varying external magnetic field, the sensor magnetization and the particle magnetization, so the relative magnetization direction of both particle and sensor are the same independent of time.

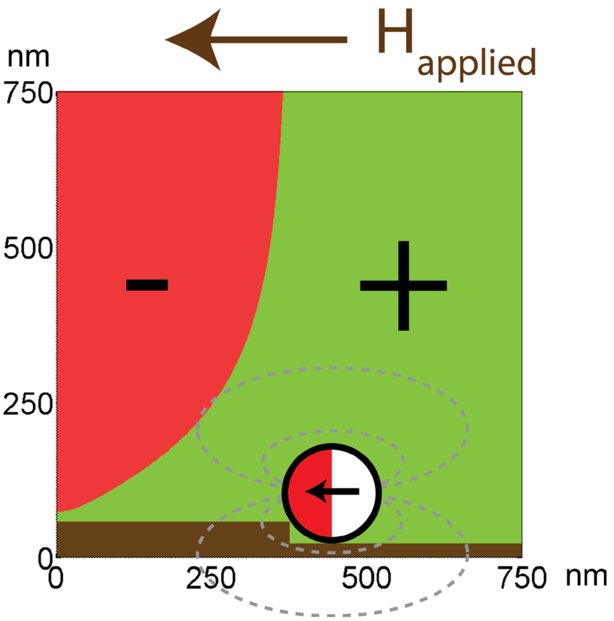

Figure S1: Simulation of sensor signal sign of magnetic resistance change due to point particle

The sign of the signal (change of sensor magnetoresistance) due to a single particle is plotted here from a Maxwell simulation. The signal sign is calculated as the sign of the scalar product of both superposition field and GMR

sensor stray field from the simulation results. It is calculated over the symmetric half unit cell (GMR sensor strip and trench) and plotted up to 750 nm. The negative and positive regions continue to be separated by a vertical line above the unit cell shown here. The signal magnitude (which decays with distance) is not plotted here for easier illustration of signal sign. Particles located in red colored regions give rise to a negative sensor signal (decrease in magnetoresistance), and particles located in green regions give rise to a positive signal (increase in magnetoresistance). A particle located partly in red and green regions has parts of the particle that give rise to both a positive and a negative signal. This graphic is not intended for signal quantitation but for showing regions where particles contribute to positive and negative signal. A single MagCellect particle with schematic field lines penetrating the sensor is plotted for size comparison.

Generally particles located above the trench give rise to a positive signal. Particles are magnetized by the local magnetic field which is in the direction of the externally applied magnetic field. The particle magnetization is parallel to the stray field of the sensor and thus the signal is positive.

Generally particles located above the sensor strip (and not directly at the surface of the sensor), give rise to a negative signal. The particles are magnetized by the local magnetic field which is in the direction of the externally applied magnetic field. The sensor stray field here is antiparallel to the particle magnetization which yields the negative signal sign.

Very close to the sensor strip (~30 nm above the center, larger at the edge) the signal is positive. The stray field of the sensor is in this proximity region larger than the externally applied magnetic field. Thus particles are magnetized by and therefore in the direction of the stray magnetic field, which makes the signal positive.

Properties of the magnetic particles used in our experiments

Table S1: Particle properties.

	MACS	MagCellect	Adembeads
Nominal diameter $[nm]$	50	150	300
Measured [19] $[nm]$	46 ± 13	n/a	282 ± 12
χ	2.08	5.55	1.57
Magnetization $[A/m]$	$2.47 \cdot 10^4$	$1.08 \cdot 10^5$	$5.29 \cdot 10^4$
Saturation field $[A/m]$	$1.77 \cdot 10^5$ ($1.23 \cdot 10^3 Oe$)	$2.47 \cdot 10^5$ ($3.10 \cdot 10^3 Oe$)	$2.41 \cdot 10^5$ ($3.03 \cdot 10^3 Oe$)
Saturation Moment $[Am^2]$ (single particle)	$1.61 \cdot 10^{-18}$	$1.90 \cdot 10^{-16}$	$7.48 \cdot 10^{-16}$
Measured concentration (particles as supplied) $[particles/mL]$	$2 \cdot 10^{12}$	$2 \cdot 10^{11}$	$1.8 \cdot 10^{11}$
Used concentration $[particles/mL]$	$2 \cdot 10^{12}$	$6 \cdot 10^{10}$	$1.2 \cdot 10^{10}$

Flowrates and velocity gradients

A flowrate range from 10 uL/min down to 0.1 uL/min translates, with the given microfluidic geometry (width=200 um, height=50 um), to microscopically relevant velocity gradients at the sensor surface of $2{,}700/sec$ down to $27/sec$. This velocity gradient at the surface is important (and not the flowrate per se) as it translates proportionally into the shear flow and related particle shear force. For a wide channel at the bottom surface the velocity gradient is linearly related to the flowrate.

Supplement to Figure 3

In Figure 3 (d,e,f) magnetic adhesion (3 sigma) noise curve slopes curves upwards for flowrates from 2 – 10 uL/min. This is an artifact since the high flowrates were measured first (compared to curves a,b,c), first 10 uL/min, then 5 uL/min, then 2 uL/min, etc..

In this initial time period (10 uL/min), the nonspecific binding signal is rising, from no particles (0 ppm) at the beginning to some particles (~10s of ppm), as there is some – although very limited – biological nonspecific binding. This approach of the steady state leads to the artifactual upward sloping curves in the magnetic adhesion, which slightly underestimates then the SNR for high flowrates.

In Figure 3 d,e,f we plot the magnetic adhesion noise estimate instead of the total magnetic adhesion as a more realistic figure for magnetic bioassays. Using reference sensors in biological assays like the BSA sensors the particle adhesion can be measured and if accurately determined, the adhesion effect can be removed from a bioassay sensor simply by subtracting the BSA particle adhesion reference signal. However this signal subtraction yields an uncertainty on the order of the standard deviation of the reference sensor signal.

Signal to noise estimates

SNR estimates are obtained from Figure 3 (d,e,f) by dividing the specific binding by the magnetic adhesion (3 times standard deviation).

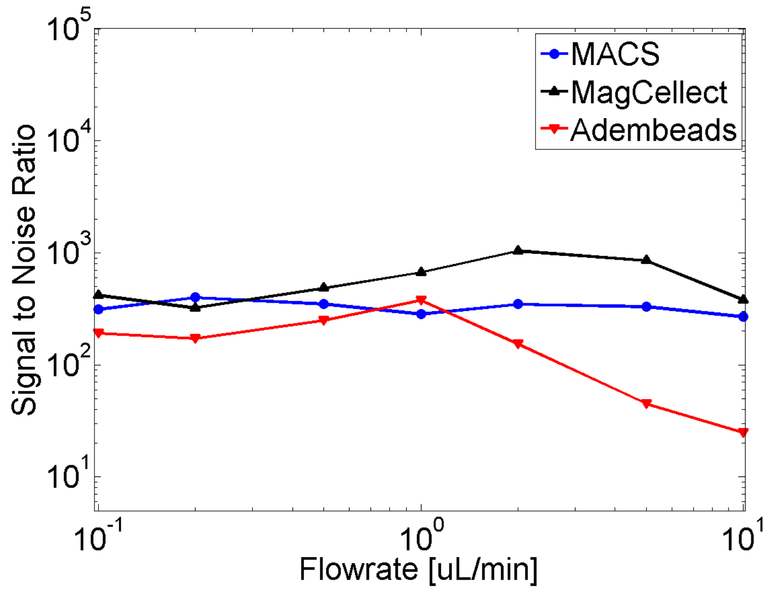

Figure S2: Signal to noise ratio estimate for different particles

SNR varies with flowrate considerably for Adembeads and less so for MagCellect particles. The overall maximum of the SNR estimate is reached for MagCellect particles at 2 uL/min.

Supplement to Figure 4

We acquired Scanning Electron Microscope images for all particles (MACS, MagCellect and Adembeads, see figure S3).

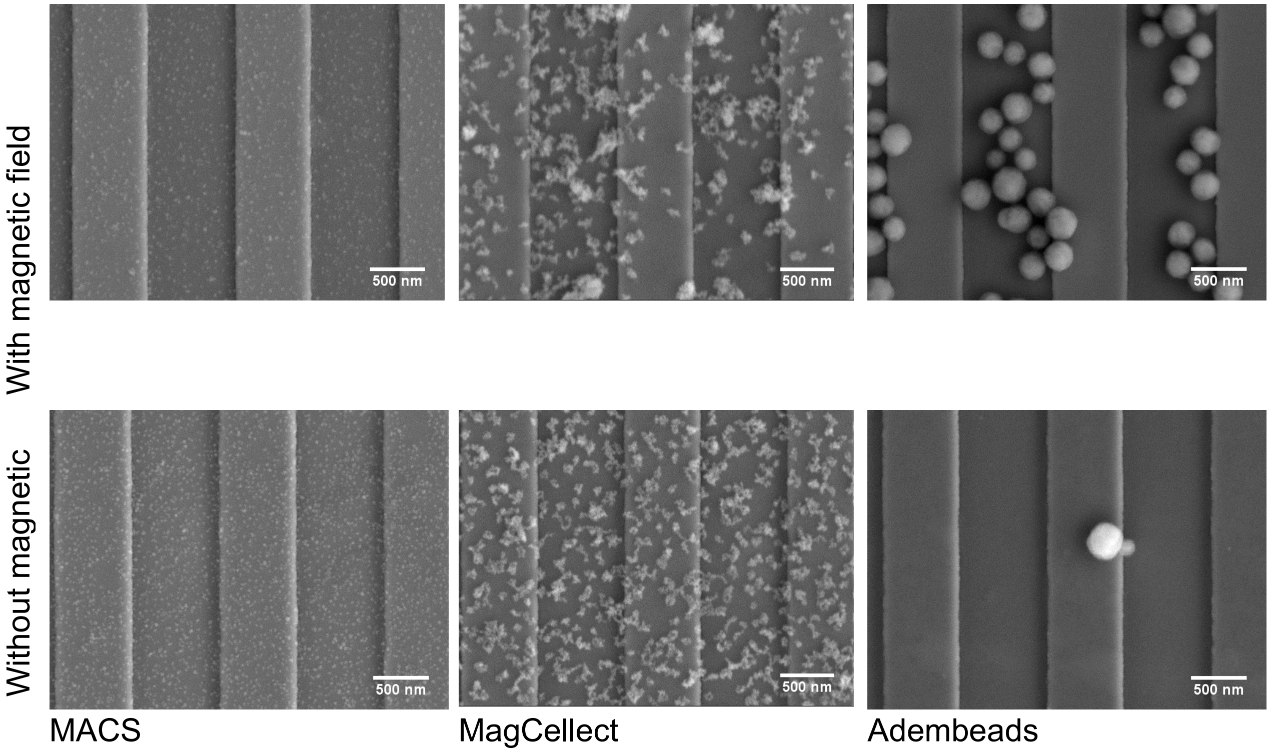

Figure S3: Scanning Electron Microscope images of magnetic nanoparticles (bright spots) after specific binding to a biotin functionalized sensor area. The different sizes of particles binding on the sensor are clearly distinguishable. The images were acquired after MACS, MagCellect or Adembeads particles specifically bound to the sensor under an applied AC magnetic field (top row) or without magnetic field (bottom row). Generally the binding locations were more uniform without magnetic field. For Adembeads, the magnetic field led to larger numbers of bound particles.

From these images the particle coverage ratios were extracted (see Figure S4).

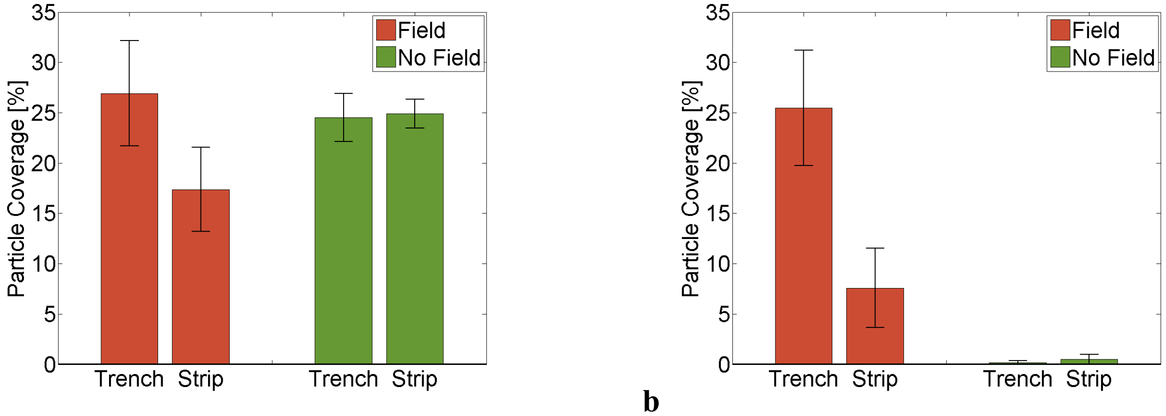

a b

Figure S4: Particle Coverage Ratios (in % of total area) extracted from SEM images of specifically bound (a) MagCellect and (b) Ademtech particles for sensor trench and strip locations for cases with applied magnetic field or without field. Particle coverage was more uniform without magnetic field. No reproducible automatic measurement was possible for MACS particles due to blurring with background due to their small size compared to the chosen GMR sensor strip and trench area.

Force scaling

To get a scale for the flow force needed to wash particles off the surface, Stokes drag forces for surface adhering particles modelled as solid spheres are 0.2 pN for 300 nm diameter (Adembeads) particles and 0.05 pN for 150 nm diameter (MagCellect) at 1 uL/min. Due to the high ionic strength of the PBS buffer, double layer effects increasing the effective size of the particles are negligible.

Segre-Silberberg effect

A Segre-Silberberg effect that could potentially concentrate particles in certain locations is negligible as the particle diameter to channel dimension is very small [1].

Supplement to Figure 5

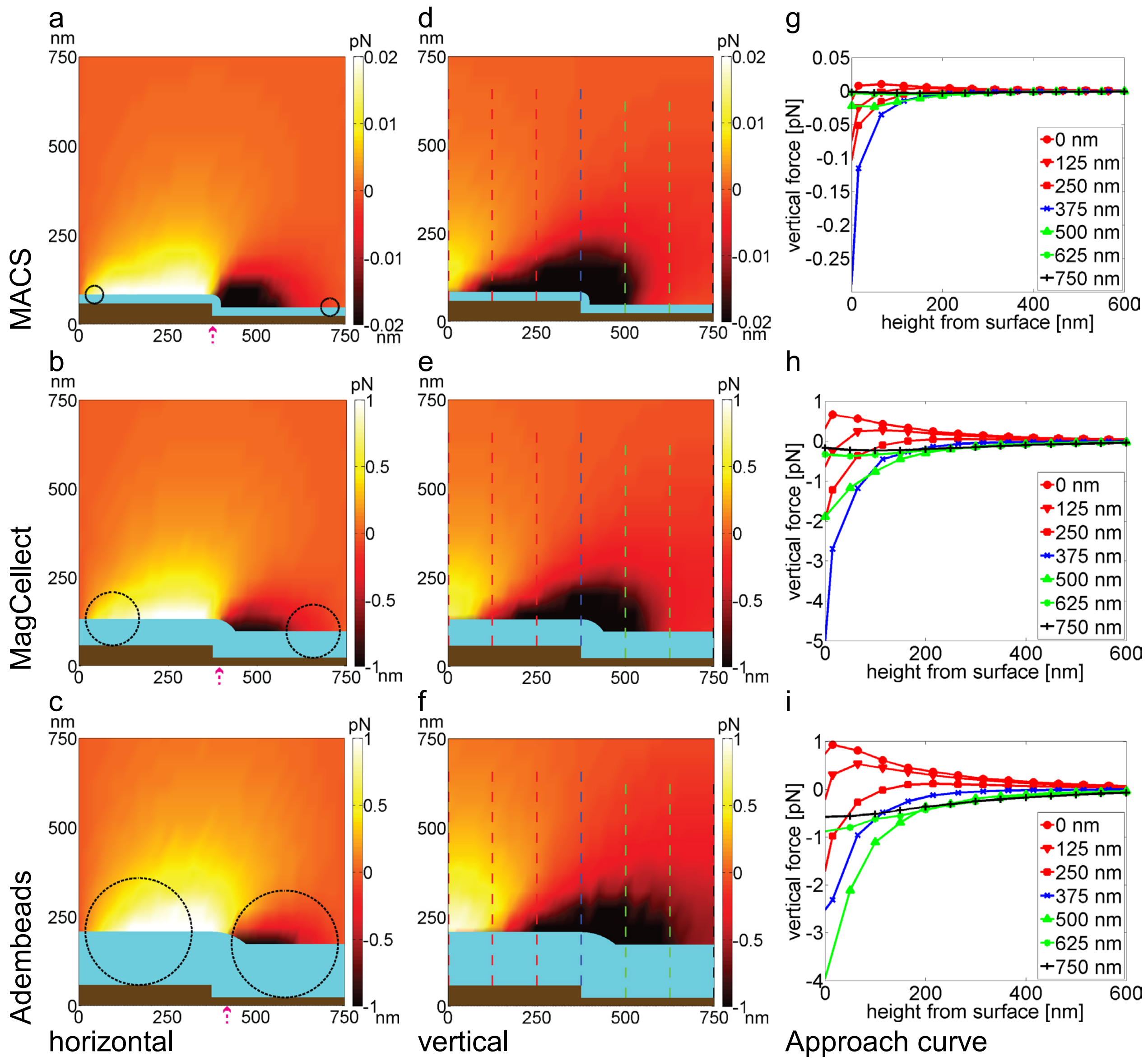

Figure S5: Plots of (a,b,c) horizontal and (d,e,f,g,h,i) vertical magnetic forces acting on the different nanoparticles

Forces are plotted in a cross-section of half of a unit sensor strip / trench cell (axisymmetric to the left and the right plot border). The sensor strip and trench region are colored brown. The vertical direction is perpendicular to the sensor surface and the positive horizontal axis is along the flow direction. Particles are modeled as Langevin spheres and the force is calculated at the center of the particle (details in methods). The finite size of the particles leads to forbidden particle locations - one particle radius or less away from the sensor - colored light blue. An AC magnetic field is applied, so the magnetization of the magnetic nanoparticles and magnetization of the GMR sensor change

over time. Calculated forces for maximum magnetic field amplitude (for 50 Oe) are shown here, with the time-averaged force at half this value. Positive values denote forces in positive x or y direction (to the right in horizontal and upwards in vertical plot).

(a,b,c) Opposing horizontal forces push particles from the sensor strip top to the trench edge and from sensor trench to trench edge. Dotted circles are shown for particle size comparison only. The zero force equilibrium point for the horizontal magnetic force is not directly at the sensor edge but moved from there inwards into the trench (see arrow under images). The equilibrium point is moved inwards further the larger the particle is.

(d,e,f) The vertical forces on the particles are attractive above the trench (with particles magnetized the same direction as the field gradient) and repulsive over the sensor strip close to the sensor surface (with particles magnetized by the applied field which is opposing the direction of the sensor stray field gradient – see supplemental figure S1).

(g,h,i) Approach curves show consistently attractive forces for particles close to trench edge but a repulsive force barrier of about 0.4 pN over a distance of 200 nm for particles approaching over the sensor stack for Adembeads and MagCellect and much lower force barriers on the order of 0.001 for MACS particles.

Supplement to Figure 6

As mentioned in the introduction, MagCellect particles show a great amount of magnetic adhesion to the sensor if no flow is applied. Thus, when performing assays without flow, the particles adhere to the sensor and get magnetically trapped, so that no useful biological data can be extracted. Figure S6 shows the binding/adhesion curves for protein, biotin-BSA and BSA sensors of a biological assay with flow (and low magnetic adhesion) compared to binding/adhesion curves without flow (and large magnetic adhesion).

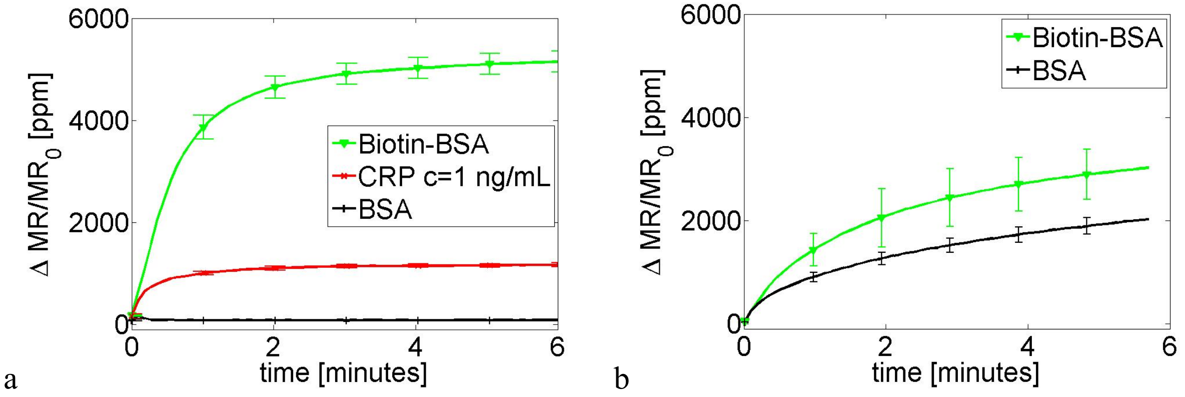

Figure S6: Real time binding and adhesion curves of MagCellect particles to sensor with microfluidic flow (a) and without microfluidic flow (b). (a) Using microfluidic sample delivery at flowrate of 2 uL/min the magnetic adhesion to the sensors is suppressed (low BSA binding magnitude and standard deviation), while a high positive control signal (biotin-BSA) is recorded, yielding a high dynamic range for protein assays as shown in Figure 6. A curve for CRP at 1 ng/mL is shown here. (b) In an open well approach – i.e. without microfluidic flow, MagCellect particles show a great amount of magnetic adhesion to the sensor. The biotin-BSA signal is suppressed compared to microfluidic experiments and is only somewhat larger as the BSA signal. Both signals' standard deviations are increased compared to the microfluidic case. This renders this non-microfluidic approach not suitable for protein assays, as the magnetic adhesion yields increased signal variations, and with increased background signal and suppressed positive control signal a much lower dynamic range. Error bars denote sample standard deviation.

These figures show that microfluidics increase the particle specificity, enabling a large dynamic range and low background signal. Without microfluidics particles adhere magnetically, non-specific to the BSA surface and have a hugely decreased signal magnitude operation range with increased magnetic particle induced variations. Thus without microfluidics no meaningful biological experiments for MagCellect particles could be performed.

Supplement to Methods: Extracting the temporal asymptotic value

The specific binding signal is fitted using MATLAB for each flowrate (time points in the sensor signal relating to each flowrate) to the exponential function $A \cdot \left(1 - e^{-B(t-t_0)}\right) + C$ with fitting parameters A,B and C, time variable t and start time t_0. This method demonstrates excellent fitting for the individual flowrate regions of a single RnD MagCellect binding curve and extraction of two temporal asymptotic values for 2 and 5 uL/min in Figure S7.

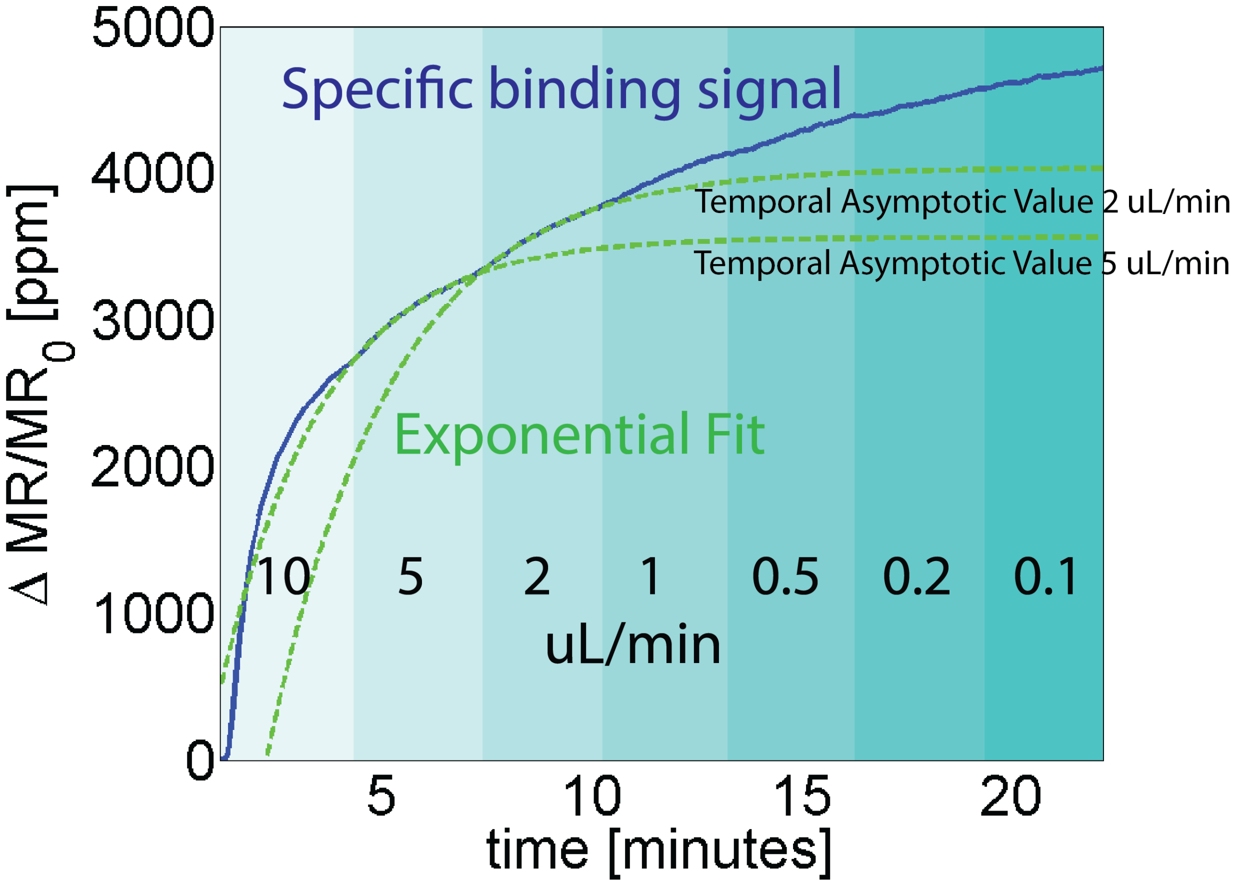

Figure S7: Illustration of temporal asymptotic value extraction. Each flowrate segment of the binding curves shows a good fit to the exponential fitting function. This allows easy extraction of the temporal asymptotic value.

[1] G. Segre and A. Silberberg, “Behaviour of macroscopic rigid spheres in Poiseuille flow,” *J. Fluid Mech.*, vol. 14, pp. 115–135, 1962.
